# Controlling Nutritional Status (CONUT) score is associated with increased rates of mortality and postoperative complications following hip fracture surgery: a propensity-matched analysis

**DOI:** 10.1007/s00590-026-04760-8

**Published:** 2026-04-24

**Authors:** Kyle Stump, Ty Langford, Nicolas Echeverria, Saqib Rehman

**Affiliations:** 1 Lewis Katz School of Medicine at Temple University, Philadelphia, USA; 2 Department of Orthopaedic Surgery and Sports Medicine, Temple University Hospital, Philadelphia, USA

**Keywords:** Malnutrition, Protein-calorie malnutrition, Controlling Nutritional Status (CONUT), Hip fracture, Orthopaedic trauma

## Abstract

**Introduction:**

Controlling Nutritional Status (CONUT) score has previously been linked to increased mortality following hip fracture. The objective of this study was to evaluate the association between preoperative CONUT score and other postoperative complication rates in patients who underwent surgical hip fracture fixation.

**Materials and methods:**

This study utilized the TriNetX Research Network to identify patients aged ≥ 65 with a previous history of hip fracture surgery between May 1, 2015, and May 1, 2025. Patients with a documented history of primary malignant neoplasm of the lower limb or secondary bony metastasis were excluded. Next, patients were separated according to calculated CONUT score using laboratory data available within six months of the index procedure. Patients with a CONUT score between 0 and 4 were considered no to low risk whereas those with a value between 5 and 12 represented the moderate to severe risk cohort. Propensity score matching was performed 1:1 based upon demographic characteristics and medical comorbidities. Outcomes were assessed within 30 days of surgery utilizing risk ratios (RR) and 95% confidence intervals (CI).

**Results:**

A total of 1,209 matched pairs were included in the analysis. After matching, there were no differences between the two groups with respect to any of the covariates included in the propensity score. CONUT score ≥ 5 was associated with elevated risk of all-cause mortality within 30 days (10.0% versus 3.8% in low to no risk group, *p* < 0.001) alongside increased incidence of pneumonia, sepsis, acute respiratory failure, acute renal failure, postoperative infection, and all-cause hospital readmission.

**Conclusion:**

CONUT score ≥ 5 is associated with increased incidence of mortality and significant postoperative complications within the 30 days following hip fracture fixation. Thus, it appears that CONUT score may be appropriate for the identification of high-risk hip fracture patients so appropriate prophylactic measures can be taken.

**Supplementary Information:**

The online version contains supplementary material available at 10.1007/s00590-026-04760-8.

## Introduction

 Hip fractures carry substantial short-term mortality and long-term functional decline, therefore necessitating optimal perioperative risk stratification for improving outcomes after surgical fixation [1–4]. Hip fractures are a global health problem, most commonly affecting adults in their eighth decade of life or later [5, 6]. Although the incidence of these events is projected to drop by 2050, their prevalence is expected to rise 4.5 times due to an aging world population [7, 8]. In addition to the patient’s medical needs, hip fractures create hospital and caregiver issues due to prolonged hospital stays, rehabilitation requirements, and complication risk [1–4]. Additionally, malnutrition is common in this aging population and strongly associated with adverse events following hip fracture repair, including delayed wound healing, infections, readmissions, delirium, declined mobility, and death; but routine clinical assessment is inconsistent [1–4]. Consequently, there is a need for pragmatic, validated tools that capture the multidimensional nature of nutritional risk and can be readily operationalized from data available in the electronic health record.

The Controlling Nutritional Status (CONUT) score is a composite index that incorporates serum albumin, total lymphocyte count, and total cholesterol to estimate global nutritional status, yielding a single numerical score on 0–12 scale with higher values indicating worse nutritional status [9]. Since it can be calculated from routinely obtained laboratory values, CONUT is attractive for real-time risk assessment. Prior work has linked elevated CONUT scores to increased mortality after hip fracture, suggesting prognostic value in this setting [10]. However, the relationship between preoperative CONUT category and a broader spectrum of postoperative complications including infectious, respiratory, renal, thromboembolic, and surgical-site events after operative hip fracture repair remains incompletely defined. Moreover, many existing studies are limited by single-center designs, small sample sizes, and incomplete adjustment for confounding.

To address these gaps, we used a large, multicenter electronic health record network to evaluate whether preoperative CONUT score is associated with 30-day mortality and major postoperative complications after surgical fixation of hip fractures. We applied propensity score matching to balance demographics and comorbidities between nutritional-risk groups, enabling a robust assessment of associations beyond mortality alone. Based on prior literature, we hypothesized that CONUT ≥ 5 (indicating moderate to severe risk) would be associated with higher risks of death and postoperative complications compared with CONUT 0–4 (no to low risk) [11]. Establishing the prognostic utility of CONUT score for a wide range of clinically meaningful endpoints could inform targeted perioperative optimization and post-discharge planning for high-risk patients. We hypothesize that patients with a preoperative CONUT score ≥ 5 will experience higher rates of postoperative complications following operative hip fracture repair.

## Materials and methods

### Data source

The study employed the TriNetX Research Network database (Cambridge, Massachusetts), a global healthcare data network that compiles real-time, de-identified electronic medical data from over 120 healthcare organizations (HCOs) and 250 million patient lives [12]. Participating HCOs permit the use of their information in exchange for access to the platform’s research capabilities at no additional cost, which includes clinical trial protocol design, database query, and drug safety analysis.

### Ethical approval

The TriNetX Research Network is compliant with the Health Insurance Portability and Accountability Act of 1996 (HIPAA) Security Rule and certified according to the ISO 27001:2013 standard. For these reasons, the study conferred minimal risk to patient privacy and was exempt from institutional review board approval and informed consent. This study was performed and reported in accordance with Strengthening the Reporting of Observational Studies in Epidemiology (STROBE) guidelines.

### Cohort selection

**Fig. 1:**
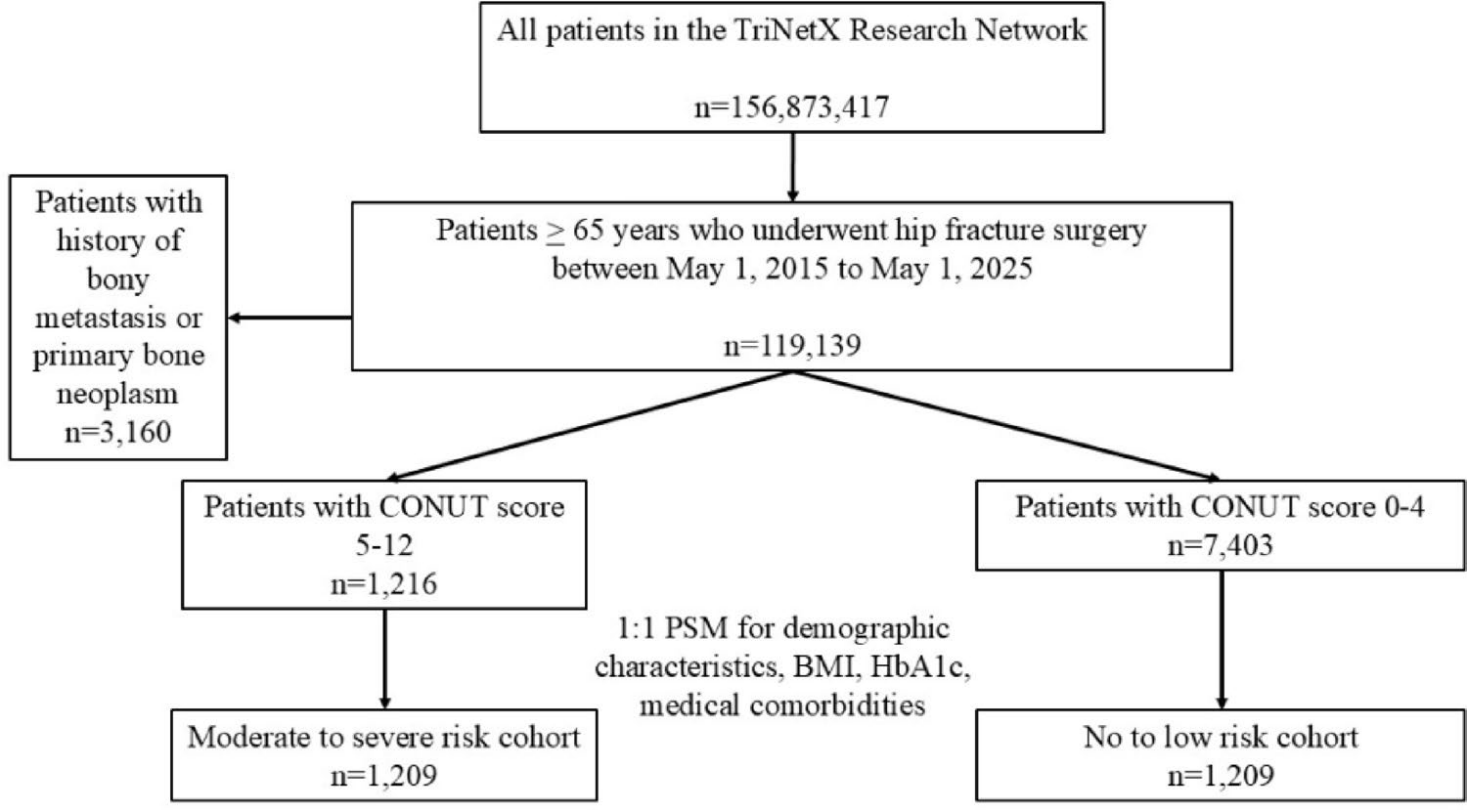
Patient selection and cohort design. N = number of patients. *Abbreviations*: CONUT, Controlling Nutritional Status; BMI, body mass index; HbA1c, hemoglobin A1c; PSM, propensity-score matching

All data included in this analysis were obtained on September 6, 2025, from the TriNetX Research Network database within the 10-year period beginning May 1, 2015, and ending May 1, 2025. Inclusion criteria, exclusion criteria, propensity score matching criteria, and study endpoints utilized classifications outlined by the International Classification of Diseases, Tenth Revision, Clinical Modification (ICD-10-CM) codes and Current Procedural Terminology (CPT). Patient cohorts were first established by querying the TriNetX database for all patients aged 65 years and older who underwent operative hip fracture repair (CPT 27235, 27236, 27244, or 27245). Patients with a documented history of primary malignant neoplasm of the lower limb or secondary bony metastasis were excluded. Next, patients were separated into two groups according to laboratory data available within six months of the index procedure (Supplemental Table 1). Patients with a serum albumin ≥ 3.0 g/dL, serum lymphocytes ≥ 1200/µL, and total serum cholesterol ≥ 140.0 mg/dL represented the no to low nutritional risk group, corresponding to a CONUT score between 0 and 4 [13]. Patients with serum albumin < 3.0 g/dL, serum lymphocytes < 1200/µL, and total serum cholesterol < 140.0 mg/dL were allocated to the moderate to severe risk cohort, reflecting a CONUT score between 5 and 12 [13].Patients without complete serum albumin, cholesterol, and total lymphocyte count within the six month preoperative period or who did not exactly satisfy these categories were excluded. The initial query yielded 1216 patients in the moderate to severe risk group and 7403 patients in the no to low risk group (Fig. 1).

### Propensity score matching

Next, the two cohorts were prepared for 1:1 propensity matching to limit the potential for confounding covariates. The characteristics chosen for matching included age at index, sex, race, ethnicity, body mass index (BMI), hemoglobin A1c (HbA1c), essential hypertension (ICD-10-CM I10), chronic kidney disease (ICD-10-CM N18), osteoporosis without current pathological fracture (ICD-10-CM M81), diabetes mellitus (DM) (ICD-10-CM E8-E13), chronic lower respiratory diseases (ICD-10-CM J44), liver disease (ICD-10-CM K76), nicotine dependence (ICD-10-CM F17), hyperlipidemia (ICD-10-CM E78.5), and long term systemic steroid use (ICD-10-CM Z79.52). Patients who lacked data for any of the matching criteria were excluded from the analysis. Successful cohort matching was reflected by a standard difference less than or equal to 0.1 for each of the selected covariates.

### Outcomes of interest

Primary outcomes included mortality, pneumonia, venous thromboembolism (VTE), sepsis, acute respiratory failure, acute renal failure, postoperative infection, emergency department (ED) utilization, and all-cause hospital readmission (from postoperative day five). All outcomes were assessed within 30-days following hip fracture repair.

### Statistical analysis

Baseline characteristics were evaluated using descriptive statistics. Binary outcomes for each endpoint were assessed with relative risk (RR) and 95% confidence intervals (CI). Kaplan-Meier (K-M) curves and Cox proportional hazards modeling were used to estimate postoperative mortality and determine hazard ratios. All statistical procedures were automatically performed within the TriNetX research platform. A two-sided *P*-value < 0.05 was considered to be statistically significant.

## Results

A total of 1209 pairs were included in this analysis (Table 1). The average age at index after propensity score matching was 78.6 ± 8.4 years in the moderate to severe risk group compared with 78.8 ± 8.8 years in the no to low risk group. Approximately 51.4% of the CONUT 5–12 cohort was female whereas female patients represented 50.2% of the CONUT 0–4 cohort. The majority of patients were White, representing 76.9% and 76.3% of the CONUT 5–12 group and CONUT 0–4 group, respectively, while Black or African American patients comprised approximately 7.0% of each cohort.

The most prevalent comorbidity was hypertension, co-occurring in 87.8% and 88.8% of the CONUT 5–12 and CONUT 0–4 cohorts, respectively (Table 1). Nearly 30% of both groups had a concomitant diagnosis of osteoporosis. After propensity score matching, rates of all other medical covariates assessed were similar between the two groups. The mean BMI did not differ between the two groups. BMI averaged 24.7 ± 5.6 kg/m^2^ in the moderate to severe risk group compared with 25.2 ± 5.6 kg/m^2^ in the no to low risk group (Table 1). The mean HbA1c was 6.2 ± 1.7% in the CONUT 5–12 group compared with 6.4 ± 1.8% in the CONUT 0–4 group, which varied significantly after matching (std 0.131). However, the distribution of patients across all HbA1c strata was similar (Table 1).

**Fig. 2:**
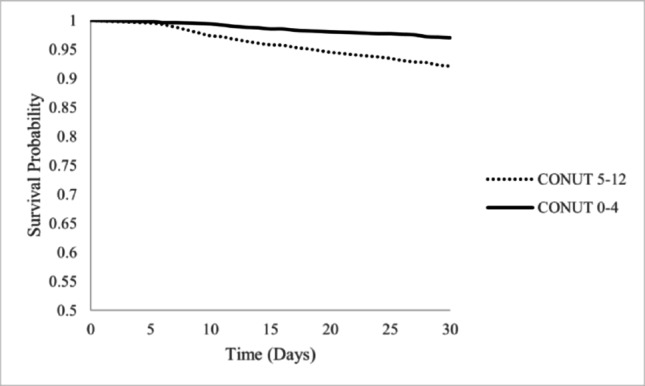
Thirty day mortality Kaplan Meier curve. Solid line denotes no to low risk of malnutrition (CONUT 0–4). Dotted line denotes moderate to severe risk of malnutrition (CONUT 5–12). *Abbreviations*: CONUT, Controlling Nutritional Status

CONUT score ≥ 5 was associated with significantly elevated risk of 30-day mortality according to Kaplan-Meier Cox Proportional modeling (10.0% versus 3.8%, HR 2.748, 95% CI 1.950–3.875, *p* < 0.001) (Fig. 2).

In addition, patients in the moderate to severe risk cohort experienced significantly increased risk of other postoperative complications within 30-days (Table 2). The incidence of postoperative pneumonia was approximately 7.5% in the CONUT 5–12 group compared with 5.0% in the CONUT 0–4 cohort (RR 1.492, 95% CI 1.089–2.043, *p* = 0.012). Similarly, the rate of sepsis was more than doubled in the moderate to severe risk group (RR 2.071, 95% CI 1.446–2.968, *p* < 0.001). CONUT score ≥ 5 was also associated with elevated risk of acute respiratory failure (RR 1.500, 95% CI 1.120–2.008, *p* = 0.006) and acute renal failure (RR 1.267, 95% CI 1.055–1.522, *p* = 0.011). Postoperative infection rates were relatively low, occurring in only 2.6% of patients in the moderate to severe risk group compared with 0.8% of those in the no to low risk cohort (RR 3.200, 95% CI 1.580–6.480, *p* = 0.001). Lastly, CONUT score ≥ 5 was associated with elevated risk of all-cause hospital readmission (RR 1.215, 95% CI 1.122–1.316, *p* < 0.001). Rates of all other complications assessed were similar between the two groups.

**Table 1:** Baseline characteristics and propensity score matching outcomes

Covariate	Before matching				After matching			
	Moderate to severe risk (CONUT 5–12)	No to low risk (CONUT 0–4)	*P*-value	Std diff	Moderate to severe risk (CONUT 5–12)	No to low risk (CONUT 0–4)	*P*-value	Std diff
*Baseline demographics*								
Age at index	78.6 (8.4)	79.6 (8.3)	< 0.001	0.129	78.6 (8.4)	78.8 (8.8)	0.540	0.025
White	937 (77.1)	6018 (81.3)	< 0.001	0.104	930 (76.9)	923 (76.3)	0.737	0.014
Female	622 (51.2)	5258 (71.0)	< 0.001	0.416	622 (51.4)	607 (50.2)	0.542	0.025
Hispanic or latino	62 (5.1)	167 (2.3)	< 0.001	0.151	59 (4.9)	55 (4.5)	0.701	0.016
Not hispanic or latino	933 (76.7)	5724 (77.3)	< 0.001	0.014	929 (76.8)	937 (77.5)	0.698	0.016
Black or African American	83 (6.8)	332 (4.5)	< 0.001	0.101	83 (6.9)	87 (7.2)	0.750	0.013
Male	503 (41.4)	1647 (22.2)	< 0.001	0.419	496 (41.0)	503 (41.6)	0.773	0.012
*Medical comorbidities*								
Essential hypertension	1067 (87.7)	6362 (85.9)	0.090	0.054	1,061 (87.8)	1073 (88.8)	0.448	0.031
Chronic kidney disease	664 (54.6)	2555 (34.5)	< 0.001	0.413	658 (54.4)	659 (54.5)	0.967	0.002
Osteoporosis	357 (29.4)	2967 (40.1)	< 0.001	0.227	357 (29.5)	354 (29.3)	0.893	0.005
Diabetes mellitus	636 (52.3)	2744 (37.1)	< 0.001	0.310	630 (52.1)	635 (52.5)	0.839	0.008
Chronic obstructive pulmonary disease	450 (37.0)	1,747 (23.6)	< 0.001	0.295	444 (36.7)	431 (35.6)	0.582	0.022
Liver disease	260 (21.4)	880 (11.9)	< 0.001	0.257	254 (21.0)	243 (20.1)	0.580	0.023
Nicotine dependence	297 (24.4)	1332 (18.0)	< 0.001	0.158	294 (24.3)	305 (25.2)	0.604	0.021
Hyperlipidemia	952 (78.3)	5652 (76.3)	0.138	0.046	945 (78.2)	946 (78.2)	0.961	0.002
Long term steroid use	110 (9.0)	387 (5.2)	< 0.001	0.149	108 (8.9)	117 (9.7)	0.529	0.026
*Anthropomorphic parameters*								
Mean BMI (kg/m^2^)	24.7 (5.6)	25.2 (5.5)	0.006	0.095	24.7 (5.6)	25.2 (5.6)	0.047	0.090
0-18.50 (kg/m^2^)	241 (19.8)	1153 (15.6)	< 0.001	0.111	240 (19.9)	241 (19.9)	0.959	0.002
18.50–24.90 (kg/m^2^)	672 (55.3)	3976 (53.7)	0.313	0.031	668 (55.3)	663 (54.8)	0.838	0.008
25.0-29.9 (kg/m^2^)	670 (55.1)	3841 (51.9)	0.038	0.064	665 (55.0)	632 (52.3)	0.178	0.055
30-34.9 (kg/m^2^)	411 (33.8)	2112 (28.5)	< 0.001	0.114	407 (33.7)	405 (33.5)	0.931	0.004
35-39.9 (kg/m^2^)	193 (15.9)	886 (12.0)	< 0.001	0.113	190 (15.7)	186 (15.4)	0.822	0.009
> 40 (kg/m^2^)	115 (9.5)	458 (6.2)	< 0.001	0.122	112 (9.3)	106 (8.8)	0.670	0.017
Mean HbA1c (%)	6.2 (1.7)	6.3 (1.6)	0.219	0.042	6.2 (1.7)	6.4 (1.8)	0.004	0.131
0-5.7 (%)	523 (43.0)	2862 (38.7)	0.004	0.089	520 (43.0)	528 (43.7)	0.743	0.013
5.7–6.4 (%)	531 (43.7)	3058 (41.3)	0.122	0.048	527 (43.6)	519 (42.9)	0.743	0.013
> 6.5 (%)	443 (36.4)	2053 (27.7)	< 0.001	0.187	439 (36.3)	453 (37.5)	0.555	0.024

Continuous variables represented as quantity (standard deviation); categorical variables represented as n (% in cohort); Std diff < 0.1 after matching reflects no difference between the two groups. *Abbreviations*: BMI, body mass index; HbA1c, hemoglobin A1c; CONUT, Controlling Nutritional Status; Std diff., standard difference

**Table 2:** Postoperative complications within 30 days of hip fracture repair

Complication	Risk in moderate to severe cohort (CONUT 5–12)	Risk in no to low cohort (CONUT 0–4)	Risk difference	Risk ratio (95% CI)	*P*-value
Pneumonia	0.075	0.050	0.025	1.492 (1.089, 2.043)	**0.012**
Venous thromboembolism	0.067	0.053	0.014	1.266 (0.921, 1.739)	0.145
Sepsis	0.072	0.035	0.037	2.071 (1.446, 2.968)	**< 0.001**
Acute respiratory failure	0.087	0.058	0.029	1.500 (1.120, 2.008)	**0.006**
Acute renal failure	0.180	0.142	0.038	1.267 (1.055, 1.522)	**0.011**
Infection	0.026	0.008	0.018	3.200 (1.580, 6.480)	**0.001**
ED utilization	0.136	0.127	0.009	1.071 (0.873, 1.315)	0.509
Readmission	0.556	0.457	0.098	1.215 (1.122, 1.316)	**< 0.001**

Risk is the proportion of the cohort with the disease. Bold face type reflects statistical significance. Readmission was calculated after postoperative day five. *Abbreviations*: CONUT, Controlling Nutritional Status; ED, emergency department; CI, confidence interval

## Discussion

CONUT score is a well-established screening tool for assessing the risk of mortality following geriatric hip fracture [10, 14, 15]. Likewise, robust associations exist between other assessments of malnutrition and incidence of postoperative complications following hip fracture [3, 11]. However, it remains unknown whether CONUT score is an appropriate tool for evaluating the risk of these other complications following operative hip fracture repair. Our study found that CONUT ≥ 5 was associated with a nearly three-fold elevated risk of mortality within the immediate postoperative period. Further, patients with moderate to severe malnutrition were nearly 50% more likely to develop pneumonia whereas the incidence of sepsis was more than doubled. Moderate to severe malnutrition was also associated with increased risk of both acute respiratory and renal failure. Postoperative infection, although relatively infrequent in absolute terms, was three times more likely in patients with CONUT score ≥ 5 alongside 21% elevated risk of all-cause rehospitalization which may reflect the severity of these other postoperative complications. In contrast, rates of both VTE and ED utilization were similar between the two groups.

Our findings are consistent with existing literature that has identified malnutrition as a risk factor for postoperative complications following hip fracture surgery. A retrospective database study by Chung et al. [11] found that severe malnutrition, as defined by serum albumin < 2.5 g/dL, was associated with a 190% elevated risk of 30-day mortality and 2.7-fold increased incidence of any major complication, which included sepsis, stroke, acute renal failure, pulmonary embolism, and acute myocardial infarction. These results closely mirror those of our study, although the 30-day mortality risk was slightly lower than that reported by Chung et al. [11] which is likely attributable to the combination of patients with moderate and severe malnutrition into a single group. Likewise, Wilson et al. [16] found that patients with serum albumin < 3.5 g/dL were more than twice as likely to develop any postoperative complication following hip fracture repair and were four times more likely to die during the postoperative inpatient admission. These observations are believed to result from the hypercatabolic state that occurs following hip fracture, as increased inflammation and tissue repair demands are exacerbated by low nutritional reserves and plasma proteins, ultimately leading to free-radical-mediated organ damage [3].

While CONUT score is a continuous variable, our findings, in conjunction with previous literature support a preoperative CONUT score ≥ 5 as an appropriate threshold for risk stratification [11]. For example, according to Rinninella et al. [17], CONUT score ≥ 5 was associated with a 52% elevated risk of prolonged length of hospitalization amongst patients admitted to an inpatient internal medicine unit. Similarly, Miano et al. [18] reported that patients with a moderate to high risk CONUT score were at increased risk of all-cause in-patient mortality sepsis even after adjusting for potential confounders on multi-variate analysis. Chen et al. [10] also employed a categorical approach to the evaluation of CONUT score in hip fracture patients and found that CONUT score ≥ 5 conferred more than a two-fold increased likelihood of one year mortality following hip fracture surgery. Particularly against the backdrop of the results of our analysis, it appears that a CONUT score of 5 is an appropriate threshold when screening hip fracture patients for malnutrition.

Nonetheless, the appropriate diagnosis of surgical patients with malnutrition remains a challenge. Although previous studies have utilized serum albumin levels to reflect nutritional status, the American Society for Parenteral and Enteral Nutrition recommends against the use of serum albumin and other negative acute phase reactants in isolationas these are heavily influenced by host inflammation in addition to nutritional status [19–21]. As a result, CONUT score is also likely reflective of both of these states. Nonetheless, its use as a screening is more sensitive for the detection of malnutrition than other criteria such as the Subjective Global Assessment and Full Nutritional Assessment Malnutrition Universal Screening Tool (MUST) [13, 22]. When compared with the gold standard for diagnosis of malnutrition in elderly patients, the Mini Nutritional Assessment (MNA), CONUT score demonstrated poor sensitivity (43%) and moderate specificity (72%) in diagnosing malnutrition and malnutrition risk [23]. This is likely attributable to the questionnaire style of the MNA that combines anthropomorphic parameters with an extensive self-assessment of dietary habits [24]. However, in the acute setting of a hip fracture, a patient may not be sufficiently oriented to accurately complete the MNA. Therefore, despite CONUT’s limitation in diagnosing malnutrition compared to the MNA, its objective nature and ready availability in the acute fracture setting underscore its utility as a screening tool in this patient population, particularly against the backdrop of our findings.

The role of nutritional supplementation for hip fracture patients has not been completely elucidated within the current literature. ASPEN guidelines currently recommend nutritional supplementation only for hip fracture patients that are malnourished or at risk of becoming malnourished [25]. However, a systematic review and meta-analysis by Avenell et al. 2016 determined there was low-quality evidence that only weakly supported the efficacy of oral malnutrition supplementation to reduce postoperative mortality [26]. Similarly, nutritional supplementation was not associated with reduced incidence of postoperative complications such as venous thromboembolism, infection, and delirium [26]. In contrast, a subsequent systematic review by Malafarina et al. 2018 found that the use of oral supplements improved overall nutritional intake and resulted in fewer postoperative complications [27]. While the efficacy of this intervention remains unclear, the results of our study suggest that CONUT score ≥ 5 may be appropriate for the identification of patients who should receive nutritional supplementation in accordance with ASPEN guidelines.

This study has several inherent limitations due to the design and use of a federated electronic health record database. First, its retrospective, observational design precludes causal claims, and thus, results can only be interpreted as associations between two variables. Although propensity-score matching was performed to balance baseline characteristics between cohorts, there is also a risk of residual confounding. Notably, variables not measured by the database, such as preoperative functional status, fragility beyond the coded medical comorbidities, living situation, and social support, likely also influence postoperative outcomes. Further, the matching process is unable to account for unmeasured, yet relevant variables such as surgeon experience, surgical technique, or postoperative compliance to rehabilitative protocols. Next, the use of TriNetX is wholly dependent upon medical billing codes, which can be used incorrectly or differently by the reporting institutions, reducing data accuracy and generalizability. The exclusive use of medical billing codes also limits the amount and type of data that can be extracted, as functional outcomes and length of inpatient hospitalization would be of interest but cannot be assessed. Additionally, the use of a de-identified database precludes the exclusion or balancing of patients based upon historical factors relevant to the index event such as polytrauma, previous ipsilateral hip surgery, long-term immunosuppressant use, and preexisting infection. The use of serum laboratory data available within six months prior to hip fracture surgery may not accurately reflect nutritional status at the time of operation. However, this may dilute the observations observed herein, making the presence of positive findings perhaps even more noteworthy. Conversely, it is unknown whether patients received nutritional supplementation in the perioperative period, which may confound results. Furthermore, under our definition of CONUT score, patients who did not satisfy the individual laboratory parameters exactly as defined would be excluded. For example, a patient with serum albumin < 3.0 g/mL but normal lymphocyte count and cholesterol would be excluded from the study population. While it is unknown how prevalent this phenomenon is nor whether it is biologically feasible, this may partially explain why only 7.2% of patients who underwent hip fracture repair during the study period were eligible for 1:1 propensity score matching. This could impair extrapolation of our results to the broader population and necessitates future investigations in which CONUT score is calculated for each patient individually.

## Conclusion

CONUT score ≥ 5 is associated with elevated risk of mortality and other major postoperative complications following hip fracture repair. Although its diagnostic value may be limited and these findings can only be interpreted within the context of a retrospective database study, CONUT score may be appropriate for the rapid, objective identification of patients at risk for malnutrition, enabling early nutritional assessment and possible intervention to occur.

## Supplementary Information

Below is the link to the electronic supplementary material.

Supplementary Material 1

## Data Availability

The datasets used and/or analyzed during the current study are available from the corresponding author on reasonable request.
