# Supplementary material for: Controlling Nutritional Status (CONUT) score is associated with increased rates of mortality and postoperative complications following hip fracture surgery: a propensity-matched analysis
Source: Eur J Orthop Surg Traumatol. 2026 Apr 24;36(1):180. doi: 10.1007/s00590-026-04760-8 (PMC13109153; doi:10.1007/s00590-026-04760-8)
Supplement: Supplementary file 1 — Supplementary Material 1 [file 590_2026_4760_MOESM1_ESM.docx]

| Laboratory Parameter | Normal | Mild | Moderate | Severe |
| --- | --- | --- | --- | --- |
| Serum Albumin g/mL) | ≥ 3.50 | 3.00 - 3.49 | 2.50 - 2.99 | < 2.50 |
| Score | 0 | 2 | 4 | 6 |
| Total Lymphocyte Count | ≥ 1,600 | 1,200 - 1599 | 800 - 1,199 | <800 |
| Score | 0 | 1 | 2 | 3 |
| Total Cholesterol (mg/dL) | ≥ 180 | 140 - 179 | 100 - 139 | < 100 |
| Score | 0 | 1 | 2 | 3 |
| Total CONUT Score | 0-1 | 2-4 | 5-8 | 9-12 |
| Nutritional Status | Normal | Mild | Moderate | Severe |

Supplemental Table 1 – Controlling Nutritional Status scoring. Abbreviations – CONUT, Controlling Nutritional Status.

Adapted from: Toyokawa G, Kozuma Y, Matsubara T, Haratake N, Takamori S, Akamine T, Takada K, Katsura M, Shimokawa M, Shoji F, Okamoto T, Maehara Y. Prognostic impact of controlling nutritional status score in resected lung squamous cell carcinoma. *J Thorac Dis.* 2017;9(9):2942-2951. doi:10.21037/jtd.2017.07.108
